# Efficacy of prophylactic cranial irradiation in patients with limited-disease small-cell lung cancer who were confirmed to have no brain metastasis via magnetic resonance imaging after initial chemoradiotherapy

**DOI:** 10.18632/oncotarget.24830

**Published:** 2018-04-03

**Authors:** Nobuaki Mamesaya, Kazushige Wakuda, Katsuhiro Omae, Eriko Miyawaki, Mie Kotake, Takumi Fujiwara, Takahisa Kawamura, Haruki Kobayashi, Kazuhisa Nakashima, Shota Omori, Akira Ono, Hirotsugu Kenmotsu, Tateaki Naito, Haruyasu Murakami, Keita Mori, Hideyuki Harada, Masahiro Endo, Takashi Nakajima, Toshiaki Takahashi

**Affiliations:** ^1^ Division of Thoracic Oncology, Shizuoka Cancer Center, Shizuoka, Japan; ^2^ Clinical Research Center, Shizuoka Cancer Center, Shizuoka, Japan; ^3^ Division of Radiation Therapy, Shizuoka Cancer Center, Shizuoka, Japan; ^4^ Division of Diagnostic Radiology, Shizuoka Cancer Center, Shizuoka, Japan; ^5^ Division of Pathology, Shizuoka Cancer Center, Shizuoka, Japan

**Keywords:** small-cell lung cancer, limited disease, prophylactic cranial irradiation, brain metastases

## Abstract

**Background:**

Prophylactic cranial irradiation (PCI) is recommended for patients with limited-disease small-cell lung cancer (LD-SCLC) who achieved good response to definitive chemoradiotherapy. However, most clinical studies lacked brain imaging scans before PCI. Our study aimed to investigate whether PCI has a survival benefit in patients who have no brain metastases (BM) confirmed via magnetic resonance imaging (MRI) before PCI.

**Results:**

Eighty patients were included in this study. Sixty patients received PCI (PCI group) and 20 patients did not (non-PCI group). OS was not significantly different between the two groups. The median OS time was 4.3 years (95% CI: 2.6 years–8.6 years) in the PCI group and was not reached (NR) (95% CI: 1.9 years–NR) in the non-PCI group (*p* = 0.542). Moreover, no differences were observed in the 3-year rates of PFS (46.2% and 44.4%, *p* = 0.720) and cumulative incidence of BM (24.0% vs. 27%, *p* = 0.404).

**Conclusions:**

Our result suggests that PCI may not have a survival benefit in patients with LD-SCLC confirmed to have no BM after initial therapy, even if patients achieve a good response to definitive chemoradiotherapy.

**Patients and Methods:**

We retrospectively evaluated patients with LD-SCLC who were confirmed to have no BM via MRI after initial chemoradiotherapy at the Shizuoka Cancer Center between September 2002 and August 2015. The overall survival (OS), progression-free survival (PFS), and cumulative incidence of BM were estimated using the Kaplan–Meier method between patients who received PCI and those who did not. Propensity score matching was used to balance baseline characteristics.

## INTRODUCTION

Small-cell lung cancer (SCLC) is a rapidly disseminating cancer accounting for 13%–15% of all lung cancers [1, 2]. Approximately two-thirds of SCLC cases are diagnosed with metastatic disease: extensive disease (ED) [1, 3], while the remaining one-third are diagnosed with limited disease (LD) curable via aggressive multimodality approach. The standard therapy for LD-SCLC is chemotherapy and concurrent thoracic radiotherapy (TRT) [4, 5]. The chemotherapy regimen consists of cisplatin (CDDP) or carboplatin (CBDCA) with etoposide [6, 7]. Although initial treatment for LD-SCLC demonstrates high response rate, most cases have recurrences within 1 year, with the brain being among the frequent sites of metastasis. More than 50% of patients develop brain metastases (BM), and approximately 45% of those who achieve complete response (CR) to initial therapy will develop BM as the only site of relapse [8]. The prognoses of patients with SCLC who develop BM during treatment are significantly poorer than those who do not develop BM [9, 10]. Therefore, prophylactic cranial irradiation (PCI) is expected to prolong overall survival (OS), and many randomized controlled trials have been conducted [8, 11–14]. However, they individually found no significant improvement in OS. Meanwhile, meta-analyses of these trials have shown that PCI improves OS in patients with SCLC who achieved a CR to initial treatment with chemotherapy [15, 16]. Auperin *et al.* [15] conducted a meta-analysis that assessed seven randomized trials that assessed the effects of PCI. Of the 987 patients, 847 patients (86%) had limited disease. They found that PCI decreased the cumulative incidence of BM (relative risk: 0.46; 95% CI: 0.38–0.57; *p <* 0.001). The relative risk of death in the PCI group was 0.84 (95% CI: 0.73–0.97; *p =* 0.01), which corresponds to a 5.4% absolute increase in 3-year survival (no-PCI group, 15.3%; PCI group, 20.7%). Based on this meta-analysis, the American Society of Clinical Oncology recommended PCI for patients with SCLC who achieve CR or partial response (PR) to initial therapy [17]. However, BM before PCI was not evaluated in most cases in these trials. Recently, a Japanese phase III randomized trial showed that PCI can reduce the incidence of BM but cannot improve the 3-year OS among patients with extensive disease-small cell lung cancer (ED-SCLC) confirmed to have no BM via magnetic resonance imaging (MRI) before receiving PCI [18]. MRI is more sensitive for detecting BM than contrast-enhanced computed tomography (CT) [19], and MRI is the best assessment tool for choosing the optimal treatment approach for BM. Therefore, whether PCI has clinical benefit or not to patients with LD-SCLC who have no BM under MRI before PCI is controversial. Our study aimed to investigate whether PCI has survival benefit in patients with LD-SCLC confirmed to have no BM via MRI after initial chemoradiotherapy.

## RESULTS

### Patient characteristics

**Figure 1:**
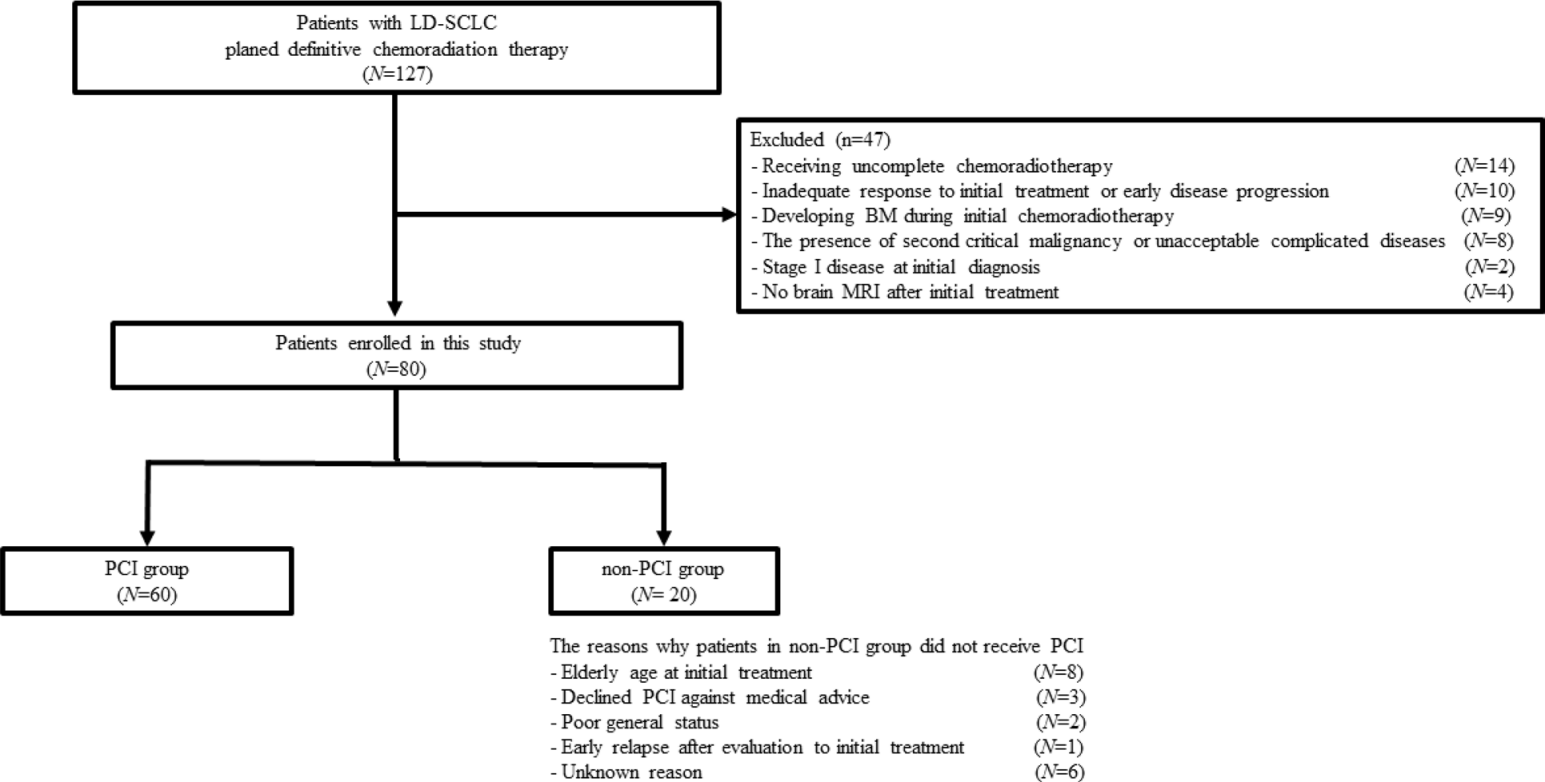
Flow diagram of the planned chemoradiotherapy for 127 patients with LD-SCLC 47 patients were excluded because of some reasons and 80 patients were enrolled in this study. They were divided into the PCI group (*N* = 60) and non-PCI group (*N* = 20). Abbreviation: LD-SCLC, limited disease-small cell lung cancer; PCI, prophylactic cranial irradiation; BM, brain metastases.

A total of 127 patients with LD-SCLC were indicated to receive definitive chemoradiotherapy. Of these, 47 patients were excluded because of developing BM during initial chemoradiotherapy (*N =* 9); receiving uncomplete chemoradiotherapy (*N =* 14); inadequate response to initial treatment or early disease progression (*N =* 10); second critical malignancy or unacceptable complicated diseases (*N =* 8); stage I disease at initial diagnosis (*N =* 2); and no brain MRI after initial treatment (*N =* 4). Finally, 80 patients were enrolled, and they were divided into the PCI group (*N =* 60) and non-PCI group (*N =* 20) (Figure 1). PCI was delivered at 25 Gy in 10 daily fractions to the whole brain. Patient characteristics are summarized in Table 1. At baseline, age, smoking history, the choice of platinum-containing drug, and the timing of TRT differed significantly between the two groups. Propensity score matching was performed as 1:1 match of the PCI and non-PCI group. After adjustment for propensity scores, 19 pairs were matched between two groups, and all covariates were well balanced among patients treated with and without PCI (Table 2). Patients in the non-PCI group did not receive PCI due to the following reasons: elderly age at initial treatment (*N =* 8); declined PCI against medical advice (*N =* 3); poor general status (*N =* 2); early relapse after evaluation to initial treatment (*N =* 1); and unknown reason (*N =* 6) (Figure 1).

**Table 1:** Patient characteristics (*N* = 80)

	Group		
	PCI (*N* = 60)	non-PCI (*N* = 20)	*p* value
Age at diagnosis, years			0.028
Median (Range)	64 (34–82)	72.5 (56–83)	
Gender			0.168
Male	43 (72)	11 (55)	
Female	17 (28)	9 (45)	
Performance status			0.210
0	37 (62)	11 (55)	
1	23 (38)	8 (40)	
2	0 (0)	1 (5)	
Smoking history			0.013
Current/Former	60 (100)	18 (90)	
Never	0 (0)	2 (10)	
Stage			0.184
IIA	4 (7)	1 (5)	
IIB	4 (7)	5 (25)	
IIIA	29 (48)	7 (35)	
IIIB	15 (25)	6 (30)	
IIIC	8 (13)	1 (5)	
Platinum-containing drug			0.002
CDDP	54 (90)	12 (60)	
CBDCA	6 (10)	8 (40)	
TRT method			<0.001
Concurrent	58 (97)	11 (55)	
Sequential	2 (3)	9 (45)	
Response to treatment			0.133
CR	23 (38)	4 (20)	
PR	37 (62)	16 (80)	

Abbreviation: PCI, prophylactic cranial irradiation; CDDP, cisplatin; CBDCA, carboplatin; CR, complete response; PR, partial response.

**Table 2:** Patient characteristics in the propensity score–matched cohort

	Group		
	PCI (*N* = 19)	non-PCI (*N* = 19)	*p* value
Age at diagnosis, years			0.429
Median	72 (56–81)	71 (56–82)	
Gender			1
Male	12 (68)	11 (58)	
Female	7 (32)	8 (42)	
Performance status			1
0	12 (63)	11 (58)	
1	7 (37)	7 (37)	
2	0 (0)	1 (5)	
Smoking history			0.487
Current/Former	19 (100)	17 (89)	
Never	0 (0)	2 (11)	
Stage			0.270
IIA	3 (16)	1 (5)	
IIB	1 (5)	5 (26)	
IIIA	10 (53)	6 (32)	
IIIB	4 (21)	6 (32)	
IIIC	1 (5)	1 (5)	
Platinum-containing drug			0.476
CDDP	15 (79)	12 (63)	
CBDCA	4 (21)	7 (37)	
TRT method			0.063
Concurrent	17 (89)	11 (58)	
Sequential	2 (11)	8 (42)	
Response to treatment			0.170
CR	9 (47)	4 (21)	
PR	10 (53)	15 (79)	

Abbreviation: PCI, prophylactic cranial irradiation; CDDP, cisplatin; CBDCA, carboplatin; CR, complete response; PR, partial response.

### Survivals and brain metastases

**Figure 2:**
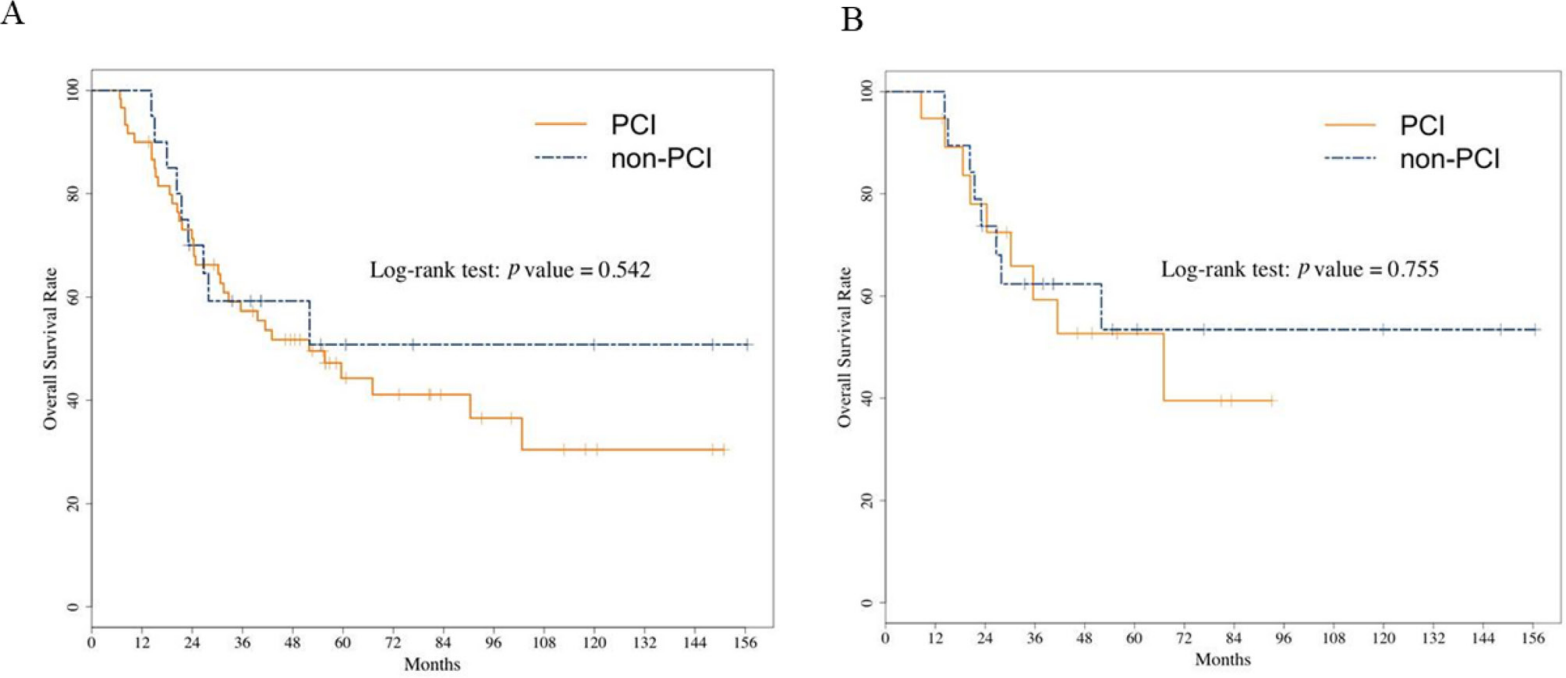
Overall survival curve in the unmatched overall cohort (**A**) and in the propensity score–matched cohort (**B**). OS was not significantly different between the PCI and the non-PCI group in the unmatched overall cohort (A). After propensity score matching, PCI was not associated with OS prolongation (B). Abbreviation: OS, overall survival; PCI, prophylactic cranial irradiation.

**Figure 3:**
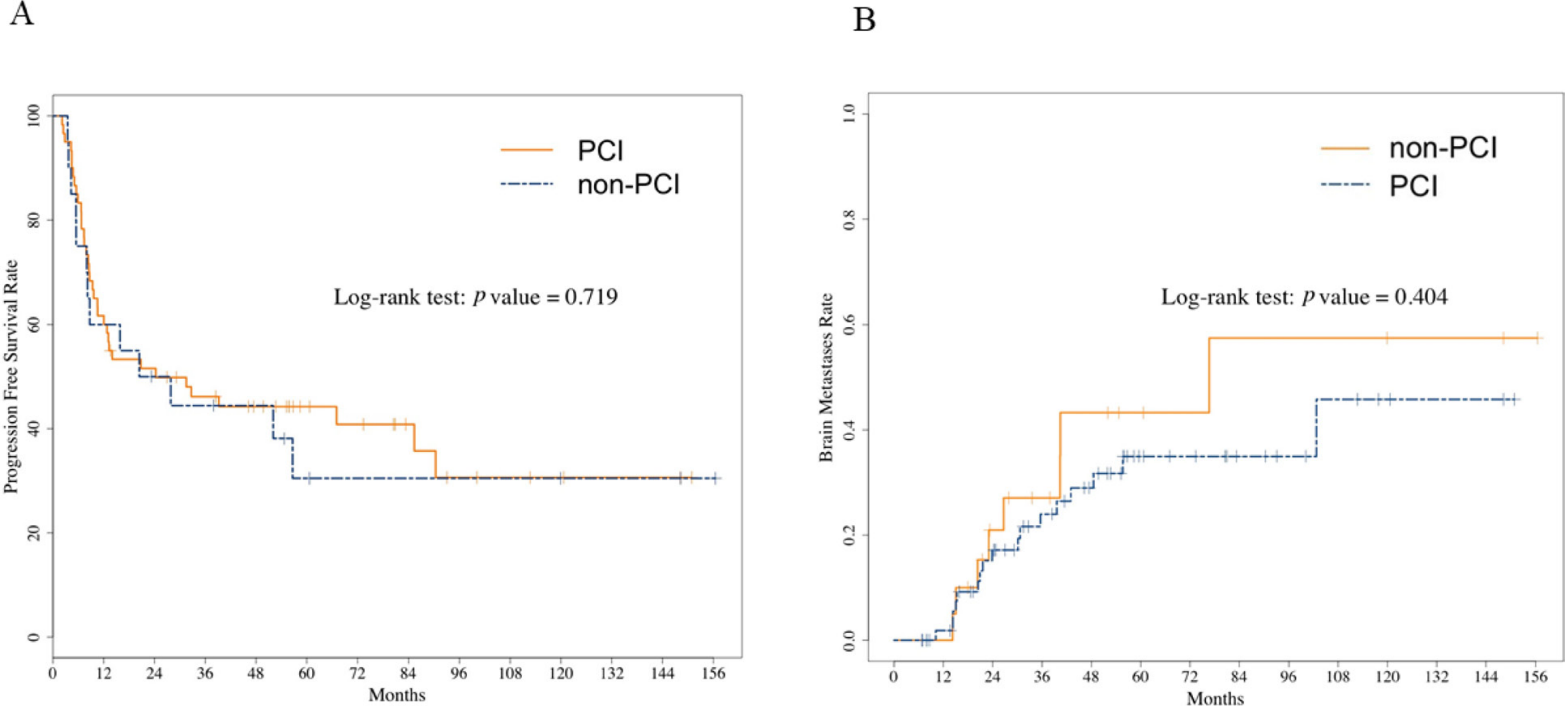
Progression-free survival (**A**) and cumulative incidence of brain metastases (**B**) in the unmatched overall cohort. No differences were observed in the PFS and cumulative incidence of BM between the PCI and the non-PCI group. Abbreviation: PCI, prophylactic cranial irradiation; PFS, Progression-free survival; BM, brain metastases.

The length of follow-up did not differ significantly between the two groups: the median was 3.4 years (range, 0.6 years–12.6 years) in the PCI group and 3.0 years (range, 1.2 years–12.1 years) in the non-PCI group (*p =* 0.608). Figure 2 shows the survival curves after initial treatment. OS was not significantly different between the two groups: the median OS was 4.3 years (95% CI: 2.6 years–8.6 years) in the PCI group and not reached (NR) (95% CI: 1.9 years–NR) in the non-PCI group (log-rank *p =* 0.542) (Figure 2A). The 1-year survival rate in the PCI and non-PCI group was 90% vs. 95%, and the 3-year survival rate was 57.3% vs. 59.2%, respectively. Supplementary Table 1 showed no associations between the patient characteristics including receiving PCI, and OS in unmatched cohort (S1). After propensity score matching, PCI was not associated with OS prolongation: the median OS was 5.6 years (95% CI: 2.5 years–NR) in the PCI group and NR (95% CI: 2.2 years–NR) in the non-PCI group (log-rank *p =* 0.755) (Figure 2B). Figure 3 shows the curves for progression-free survival (PFS) and cumulative incidence of BM. No differences were observed in the 3-year rates of PFS and cumulative incidence of BM between the PCI and the non-PCI group: PFS, 46.2% and 44.4% (log-rank *p =* 0.720); cumulative incidence of BM (24.0% vs. 27.0%, log-rank *p =* 0.404), respectively.

### Treatments after disease progression

Furthermore, second-line chemotherapy for recurrence was administered to 27 (96.4%) and 7 (70%) patients in the PCI and non-PCI groups, respectively (Table 3). Radiation therapy for BM recurrences after the initial chemoradiotherapy with or without PCI was administered to 11 (64.7%) and 6 (75.0%) patients in the PCI and non-PCI groups, respectively (Table 3). All 6 patients in the non-PCI group chose whole brain radiation therapy. All remaining patients who had BM recurrences were administered up to third- or fourth-line salvage chemotherapy without cranial irradiation for BM. Leukoencephalopathy after PCI was observed in 1 patient in the PCI group. The patient chose the best supportive care after management for leukoencephalopathy because of deteriorating Eastern Cooperative Oncology Group Performance Status (ECOG-PS) when the recurrence progressed.

**Table 3:** Treatments after disease progression

	Group	
	PCI (*N* = 60)	non-PCI (*N* = 20)
Disease recurrence^*1^	**28 (46.7%)**	**10 (50%)**
Second-line chemotherapy	27^*2^	7
Single agents	14	4
Platinum-based doublet	13	3
Third-line chemotherapy	19	6
Single agents	14	5
Platinum-based doublet	5	1
Fourth-line chemotherapy	11	5
Single agents	10	4
Platinum-based doublet	1	1
Brain metastases recurrences	17 (28.3%)	8 (40%)
Radiation therapy for BM recurrences	11	6
WBRT	2	6
WBRT + SRT	1	0
WBRT + SRS	1	0
SRT	4	0
SRS	3	0
Chemotherapy for BM recurrences	4	1
Untreated	1	0
Unknown	1	1

^*1^Brain metastases recurrences are included in disease progression.

^*2^1 patient out of the 28 who have disease recurrences received thoracic radiation therapy for local recurrence.

Abbreviation: PCI, prophylactic cranial irradiation; BM, brain metastases; WBRT, whole brain radiation therapy; SRT, Stereotactic radiotherapy; SRS, Stereotactic radiosurgery.

## DISCUSSION

PCI improves the survival and reduces the incidence of BM in patients with SCLC [15, 16, 20]. These findings indicate that PCI is recommended for LD-SCLC patients who have a good response to initial therapy. However, this recommendation was made based on studies in which brain imaging was not a standard method in initial staging or follow-up. The prevalence of detected BM during SCLC treatment was 10% in the CT era and 24% in the MRI era [21]. Manapov *et al.* [10] also reported that 13/40 (32.5%) patients with LD-SCLC who achieved CR to initial chemoradiotherapy developed BM as confirmed on contrast-enhanced cranial MRI before PCI. Of the 13 patients, 11 had asymptomatic brain metastases. These data indicate that asymptomatic BM might be missed if brain MRI is not performed after initial therapy. Therefore, we performed this retrospective study to evaluate the efficacy of PCI for LD-SCLC patients with no evidence of BM confirmed on MRI before PCI administration. PCI reduces the incidence of symptomatic BM and prolongs disease-free survival and OS in patients with ED-SCLC who responded to chemotherapy [22]. However, in this randomized trial, brain imaging was not part of standard staging and follow-up procedures, unless BM was suspected. PCI without restaging MRI is likely to result to overtreatment for patients without BM or undertreatment for patients with BM. A Japanese phase III randomized trial showed that PCI did not improve OS in ED-SCLC patients who achieved CR or PR to initial chemotherapy without confirmed BM on MRI before receiving PCI [18]. Thus, cranial irradiation as prophylaxis was shown to have no clinical benefit. In our study, a similar result regarding OS in patients with LD-SCLC who achieved CR or good PR to initial chemotherapy without BM confirmed on MRI before receiving PCI was also achieved.

However, the age, smoking history, choice of platinum-containing drug, and timing of TRT were different between patients in the PCI and non-PCI group. In general, elderly patients with lung cancer have poor prognosis [23]. Furthermore, delayed TRT with chemotherapy leads to poor patient prognosis [24, 25]. In our study, the age and frequency of sequential TRT were significantly higher in the non-PCI group than in the PCI group, but no significant difference was observed in OS. Moreover, BM incidence and survival between patients who underwent and did not undergo PCI were not significantly different if patients received adequate stereotactic irradiation and brain MRI [26].

Meanwhile, the long-term side effects of PCI are concerning. Several phase III trials showed that PCI caused a deterioration of neuropsychological and cognitive functions [27–29]. PCI is also a risk factor of leukoencephalopathy [30, 31]. Elderly patients aged >65 years had PCI-induced neurotoxicity more frequently than younger patients [32]. PCI cannot significantly improve OS or brain metastasis-free survival among elderly patients with LD-SCLC aged ≥70 years, particularly those with large tumors [33]. As such, PCI in elderly patients with LD-SCLC should be carefully considered, particularly for adequate BM management.

Our study has some limitations. First, it was a single-institutional study, and we cannot avoid selection bias. The frequency of CBDCA-based chemotherapy was higher in the non-PCI than in the PCI group. However, a meta-analysis of 4 randomized trials in patients with SCLC showed no differences in survival between CDDP and CBDCA [34]. ECOG-PS and TNM classification stage of SCLC was similar in both groups. In addition, all patients received the standard regimen for chemotherapy and TRT or PCI dose. Therefore, our study has high external validity for real-world clinical settings. Second, the study population was small, particularly that of the non-PCI group, because of the low incidence of LD-SCLC. This was because the current standard of care in patients with LD-SCLC who have a good response to initial chemoradiotherapy is to receive PCI, although no individual randomized trial with PCI has demonstrated a significant OS prolongation for patients with LD-SCLC. Only few reports on patients with LD-SCLC who received standard chemoradiotherapy without PCI are available [35–37]. However, no studies about the clinical benefits of PCI in patients with LD-SCLC confirmed with no BM on MRI have been conducted. Third, patient prognosis was better in our study than that in previous reports [38]. This may be because the current standard assessment with contrast-enhanced CT or PET/CT and MRI in pre- and post-treatment follow-up are more accurate. Recently, a Japanese randomized phase 3 study (JCOG0202) reported a median OS of 3.2 years (95% CI: 2.4–4.1) in patients with LD-SCLC treated with etoposide and CDDP plus concurrent accelerated-hyper fractionation-TRT [39]. Patient prognosis in our study was consistent with that of JCOG0202, considering our study excluded the patients with brain metastases during the initial treatment period. Finally, some cases were censored because of patients’ request to transfer to another hospital to receive best supportive care. However, the median observation period of our study was more than 3 years, and no differences were observed in both groups.

Despite several limitations, our study is valuable because this is the first study in terms of asymptomatic BM during treatment in patients with LD-SCLC receiving PCI.

## CONCLUSIONS

The results of this retrospective study suggest that PCI may not only have a clinical benefit for survival but also the BM incidence in patients with LD-SCLC confirmed with no BM before PCI, even if patients achieve a good response to definitive chemoradiotherapy. Therefore, performing PCI should be carefully considered. Further prospective studies are warranted to identify the clinical benefit of PCI in this population.

## PATIENTS AND METHODS

### Patients

We retrospectively evaluated the efficacy of PCI in patients with LD-SCLC. LD-SCLC was defined as disease confined to one hemithorax including local extension and ipsilateral hilar, bilateral mediastinal, and supraclavicular lymph node metastases, which can be encompassed by a single radiotherapy port. Patients with malignant pleural effusion, defined as ED-SCLC, were excluded. Staging was performed via CT, MRI, bone scan, and positron-emission tomography (PET)/CT. Patients with LD-SCLC who achieved CR or PR and have no BM confirmed via MRI after initial chemoradiotherapy at Shizuoka Cancer Center from September 2002 to August 2015 were assessed. Patient data, including age at diagnosis, sex, ECOG-PS, smoking history, clinical stage according to the Union International for Cancer Control-TNM 8th edition, chemotherapy regimen, TRT method, best response to initial treatment, treatment with PCI or not, and outcomes were obtained from medical records. The study was approved by the Institutional Review Board of Shizuoka Cancer Center. We provided the patients the opportunity to opt out of this study.

### Treatment

The patients were administered 4 cycles of platinum-based treatment regimens that mainly consisted of etoposide and CDDP or CBDCA. TRT was performed concurrently or sequentially with chemotherapy. The usual prescription dose was 45 Gy in 30 fractions, administered twice daily over a 3-week period (accelerated-hyper fractionation-TRT), except for 2 patients who were delivered 48–50 Gy in 24–25 daily fractions with a sequential conventional radiotherapy. Treatment response was defined as CR or PR according to the Response Evaluation Criteria in Solid Tumors criteria version 1.1 as assessed via CT. Brain MRI after initial treatment was necessary to confirm the absence of BM. Patients who showed BM on MRI after initial therapy or who did not undergo brain MRI were excluded. PCI was delivered at 25 Gy in 10 daily fractions to the whole brain, which is the standard for LD-SCLC [40].

### Statistical analysis

We compared treatment-related characteristics between the two groups using Pearson’s chi-square test or Fisher’s exact test as well as nonparametric test of medians for categorical variables and unpaired student’s *t*-test for continuous variables. The follow-up period was calculated from the end of the initial therapy to the date of death or the date last confirmed alive. The primary endpoint of this study was OS, defined as the interval between the end of the initial chemoradiotherapy and the date of death or the last follow-up visit. The secondary endpoints were PFS and the cumulative incidence of BM. PFS was defined as the interval between the end of the initial chemoradiotherapy and the date of any recurrences detected with any imaging modality, death, or the last follow-up visit. OS curves, PFS curves, and the cumulative incidence of BM were estimated using the Kaplan–Meier method, and the groups were compared using log-rank test. Patients who remained alive were censored. Univariate and multivariate analyses of OS were performed with the Cox proportional hazards regression model in the unmatched overall cohort. Propensity score matching was also used to balance baseline characteristics between the two groups. We calculated the propensity scores using a multiple logistic regression model that included age at diagnosis, chemotherapy regimen, and TRT method.

All *p* values are two sided, and a value of < 0.05 was considered statistically significant. All statistical analyses were performed using the statistical software JMP^®^13 (SAS Institute Inc., Cary, NC, USA) and R version 3.4.2. software (Institute for Statistics and Mathematics, Vienna, Austria).

## SUPPLEMENTARY MATERIALS TABLE



## Abbreviations

SCLC: small-cell lung cancer
ED: extensive disease
LD: limited disease
TRT: thoracic radiotherapy
CDDP: cisplatin
CBDCA: carboplatin
BM: brain metastases
CR: complete response
PCI: prophylactic cranial irradiation
OS: overall survival
PR: partial response
ED-SCLC: extensive disease-small cell lung cancer
MRI: magnetic resonance imaging
CT: computed tomography
PET: positron-emission tomography
ECOG-PS: Eastern Cooperative Oncology Group Performance Status
PFS: progression-free survival
