# Supplementary material for: Efficacy of prophylactic cranial irradiation in patients with limited-disease small-cell lung cancer who were confirmed to have no brain metastasis via magnetic resonance imaging after initial chemoradiotherapy
Source: Oncotarget. 2018 Apr 3;9(25):17664–74. doi: 10.18632/oncotarget.24830 (PMC5915147; doi:10.18632/oncotarget.24830)
Supplement: Supplementary file 1 [file oncotarget-09-17664-s001.pdf]

Efficacy of prophylactic cranial irradiation in patients with limited-disease small-cell lung cancer who were confirmed to have no brain metastasis via magnetic resonance imaging after initial chemoradiotherapy

SUPPLEMENTARY MATERIALS

Supplementary Table 1: The associations between the patient characteristics and OS in the unmatched overall cohort (*N* = 80)

Univariate analysis	HR	95% CI	*p* value
Age at diagnosis			
≥75 versus <75	0.71	(0.21–1.77)	0.498
Gender			
male versus female	1.56	(0.81–3.24)	0.191
Performance status			
0 versus 1–2	0.95	(0.52–1.77)	0.869
Smoking history			
Current/former versus never	0.48	(0.145–2.93)	0.358
Stage			
II versus III	0.68	(0.26–1.50)	0.361
Platinum-containing drug			
CDDP versus CBDCA	1.30	(0.59–3.43)	0.542
TRT method			
Concurrent versus sequential	1.34	(0.58–3.91)	0.523
Response to treatment			
CR versus PR	0.65	(0.33–1.23)	0.188
PCI			
with versus without	1.26	(0.63–2.79)	0.535
**Multivariate analysis**	**HR**	**95% CI**	***p* value**
Age at diagnosis			
≥75 versus <75	0.86	(0.18–4.10)	0.851
Response to treatment			
CR versus PR	0.62	(0.31–1.20)	0.158
TRT method			
Concurrent versus sequential	1.00	(0.28–4.33)	0.988
PCI			
with versus without	1.29	(0.53–3.84)	0.596

Abbreviations: OS, overall survival; HR, hazard ratio; CI, confidence interval; CDDP, cisplatin; CBDCA, carboplatin; TRT, thoracic radiotherapy; CR, complete response; PR, partial response; PCI, prophylactic cranial irradiation.
